# Long-term survival after multimodal therapy for advanced hepatocellular carcinoma: a case report

**DOI:** 10.3389/fimmu.2026.1746806

**Published:** 2026-04-14

**Authors:** Danxin Wu, Guangyue Hu, Dan Wang, Chunli Chen, Bangchun Ming, Zhiguo Luo, Tieyan Wang, Haixia Li

**Affiliations:** 1 Department of Oncology, Hubei Provincial Clinical Research Center for Precision Diagnosis and Treatment of Liver Cancer, Taihe Hospital, Hubei University of Medicine, Shiyan, Hubei, China; 2 Key Laboratory of Cancer Therapy Resistance and Clinical Translational Study, Shiyan, Hubei, China; 3 Department of Pathology, Taihe Hospital, Hubei University of Medicine, Shiyan, Hubei, China

**Keywords:** hepatocellular carcinoma, immunotherapy, multimodal therapy approach, radiotherapy, TKI therapy

## Abstract

Hepatocellular carcinoma (HCC) complicated by portal vein tumor thrombosis (PVTT) is associated with limited therapeutic options and poor survival. Immune checkpoint inhibitors, especially when combined with tyrosine kinase inhibitors (TKIs), locoregional therapies, or radiotherapy, are reshaping the management of advanced HCC; however, the optimal way to integrate these modalities, particularly in patients with vascular invasion and low programmed death-ligand 1 (PD-L1) expression, remains uncertain. Herein, we describe a 52-year-old man with advanced HCC complicated by PVTT who initially received transarterial chemoembolization (TACE), hepatic resection, and adjuvant lenvatinib but later developed postoperative recurrent disease, including a right perirenal lesion and a synchronous right subpleural metastatic lesion. He then achieved a durable complete response and long-term survival with an immunotherapy-centered regimen combining radiotherapy, lenvatinib, and tislelizumab. This case suggests that an immunotherapy-based multimodal regimen integrating TACE, surgery, TKI therapy, radiotherapy, and programmed cell death protein 1 (PD-1) blockade can achieve deep and durable remission in selected patients with advanced HCC and low PD-L1 expression.

## Introduction

Hepatocellular carcinoma (HCC) is among the most frequently diagnosed cancers worldwide and remains a major cause of cancer-related mortality (1, 2). The high mortality-to-incidence ratio reflects its poor prognosis (1, 3, 4). In Western countries, alcohol-related liver injury predominates, whereas in Asia, chronic hepatitis B virus (HBV) infection remains the leading etiology (5). Most patients are diagnosed at advanced stages, with a median overall survival (OS) of about nine months and a 5-year survival rate below 20% (5). Even with systemic therapy, symptomatic advanced HCC carries a median OS of only 12–18 months (6).

Surgical resection remains the cornerstone of curative treatment for HCC; however, postoperative recurrence is a major challenge, with more than 70% of patients relapsing within five years (7, 8). Major vascular invasion (MVI), particularly portal vein tumor thrombosis (PVTT), defines BCLC stage C disease, occurs in roughly one-third of patients at diagnosis, and reflects highly aggressive tumor biology with a median overall survival of only 2–5 months (9, 10). Although retrospective studies suggest potential benefit from surgical resection in selected patients with MVI, the prognosis remains dismal because of low resectability and high recurrence rates (11). Thus, effective adjuvant and systemic strategies are urgently needed to improve long-term outcomes.

The advent of TKIs, particularly sorafenib and later lenvatinib, marked a pivotal milestone in the evolution of systemic therapy for advanced HCC (12, 13). However, TKI monotherapy remains limited by low ORR, underscoring the need for more effective strategies. In recent years, immune checkpoint inhibitors (ICIs), particularly those targeting PD-1/PD-L1, have reshaped the systemic treatment paradigm (5). ICI-based combinations have achieved meaningful survival benefits and are now recommended as first-line options by international guidelines (14–19). Treatment beyond progression is generally safe in patients with HCC and may benefit selected individuals due to the potential for delayed responses or sustained disease stability (16).

Among these, dual-modality regimens combining ICIs with anti-VEGF/VEGFR therapies such as TKIs or monoclonal antibodies have shown strong synergistic effects (14, 20). TKIs like lenvatinib can normalize tumor vasculature, promote T-cell infiltration, thereby potentiating the activity of PD-1 blockade (21). Clinical data from the LEAP-002 trial and other studies have suggested that lenvatinib plus PD-1/PD-L1 inhibitors can improve survival compared with lenvatinib alone in unresectable HCC, with particularly encouraging results in Asian populations (22, 23). These findings support the potential of lenvatinib–ICI combinations as a promising therapeutic paradigm for advanced HCC. Furthermore, growing evidence indicates that radiotherapy combined with ICIs can exert synergistic effects on both local tumor control and distant lesions by enhancing antitumor immunity (24). In HCC, early clinical and real-world data indicate that radiotherapy combined with PD-1/PD-L1 blockade is generally safe and can achieve favorable tumor control and survival in unresectable, recurrent, or metastatic disease, supporting this approach as a valuable multimodal option (25, 26).

Here, we report a case of BCLC-C HCC with PVTT at diagnosis that developed postoperative recurrent disease, including a right perirenal lesion and a synchronous right subpleural metastatic lesion after TACE, surgical resection, and lenvatinib treatment. Subsequent incorporation of radiotherapy and PD-1 blockade into an immunotherapy-centered multimodal regimen induced a durable complete remission and prolonged survival, suggesting that even PD-L1–low “cold” advanced HCC may be rendered highly responsive through rational combination strategies.

## Case presentation

On October 7, 2021, a 52-year-old male with a 40-year history of chronic HBV infection was admitted for evaluation of elevated serum alpha-fetoprotein (AFP) levels detected during routine testing. He had taken entecavir at 0.5 mg/day irregularly during the preceding year. He was asymptomatic at presentation, without fever, chills, nausea, vomiting, chest tightness, chest pain, dyspnea, abdominal pain, or abdominal distension. Physical examination showed no jaundice, spider angioma, or palmar erythema. No superficial lymphadenopathy was detected. Cardiopulmonary examination was unremarkable. The abdomen was flat and soft, without tenderness or rebound tenderness; no hepatosplenomegaly or ascites was identified, and there was no lower-extremity edema. His past medical history included no diabetes, hypertension, coronary artery disease, or tuberculosis, no prior surgery or major trauma, no history of blood transfusion, and no known food or drug allergies. He had a 20-year smoking history, averaging approximately 5 cigarettes per day, and a history of light alcohol consumption; both had ceased 4 years earlier. Family history revealed that the patient’s mother had died of cirrhosis at the age of 80, whereas his father was deceased of an unknown cause. There was no known family history of hereditary disease or liver cancer.

**Figure 1:**
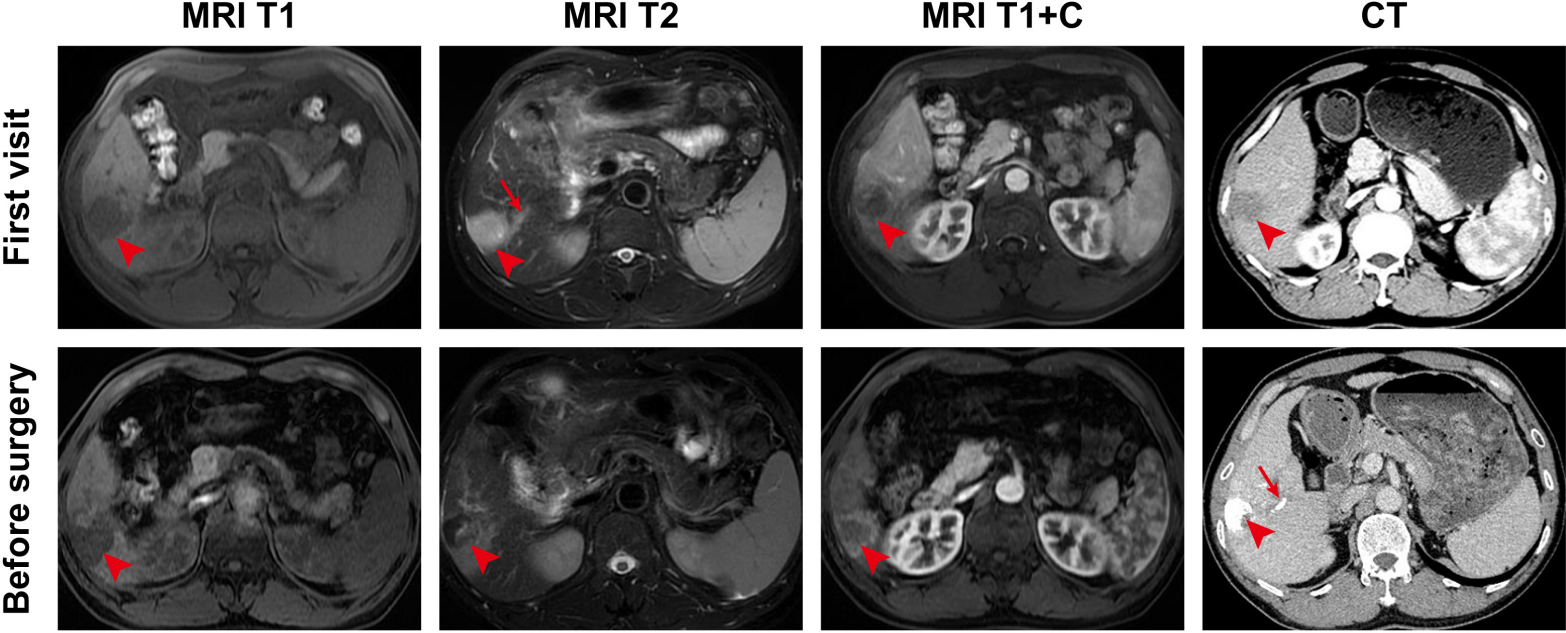
Baseline and post-TACE imaging of the primary hepatic tumor and portal vein tumor thrombus. Magnetic resonance and contrast-enhanced CT images obtained at initial diagnosis and after the first session of transarterial chemoembolization (TACE) before surgery. Panels include T1-weighted, T2-weighted, and contrast-enhanced T1-weighted MRI, as well as contrast-enhanced CT. Arrowheads indicate the hepatic tumor, and arrows indicate the portal vein tumor thrombus. CT, computed tomography; MRI, magnetic resonance imaging.

Abdominal contrast-enhanced computed tomography (CT) performed on October 8, 2021, revealed a 3.5 × 3.2 cm mass in the right anterior hepatic lobe, accompanied by splenomegaly and signs of portal hypertension. Contrast-enhanced magnetic resonance imaging (MRI) confirmed HCC with PVTT involving the right portal branch (**Figure 1**). Laboratory findings showed normal blood counts, coagulation profile, and mildly impaired hepatic function (ALT 35.3 U/L, AST 36.6 U/L, γ-GT 42.2 U/L, ALP 78.8 U/L, total bilirubin 22.9 μmol/L, and albumin 39.1 g/L). HBV DNA was <1.0 × 10² IU/mL, and AFP was 102 ng/mL (reference <13.6 ng/mL). Based on the chronic HBV background, markedly elevated AFP level, characteristic findings on contrast-enhanced imaging, and the patient’s fully preserved daily activity without cancer-related symptoms, the diagnosis was established as BCLC stage C HCC with PVTT, with Child-Pugh class A liver function and Eastern Cooperative Oncology Group (ECOG) performance status 0. A concise timeline of the patient’s treatment course and outcomes is provided in **Table 1**.

**Table 1:** Sequential treatment course and key clinical outcomes.

Time	Clinical status	Treatment	Outcome/evaluation	Adverse events
2021.10.07	Asymptomatic; elevated AFP on routine testing	Baseline work-up	BCLC stage C HCC with right branch PVTT, Child-Pugh A, ECOG 0; no extrahepatic metastasis	None
2021.10.13	Stable	TACE	Initiation of conversion treatment	None significant
2021.11.15	Stable	Post-TACE reassessment	Favorable lipiodol deposition; PVTT stable in right branch; no extrahepatic metastasis	None
2021.11.19	Resectable after reassessment	Laparoscopic partial hepatectomy	Pathology: Edmondson–Steiner III, M2 microvascular invasion, cirrhosis, intravascular tumor thrombus; negative margins	None significant
2021.12-2022.02	Postoperative recovery	Lenvatinib 8 mg/day + 2 postoperative TACE sessions	AFP normalized	Grade 1 diarrhea; later grade 2 hypertension and grade 1 proteinuria
2022.06	Right flank pain (NRS 6)	Imaging reassessment	Right perirenal and right subpleural recurrence; AFP 1,179.7 ng/mL	Cancer-related pain
2022.06-2022.07	Symptomatic recurrence	IMRT + tislelizumab (lenvatinib continued initially)	Transition to immunotherapy-based multimodal treatment	No major RT toxicity; no irAEs
2022.08	Pain resolved	Continued systemic therapy	AFP normalized; partial response	None significant
2024.04	Stable disease	Lenvatinib stopped; tislelizumab continued	Ongoing disease control	Lenvatinib discontinued for low-grade toxicities
2024.09-latest follow-up	Asymptomatic	Tislelizumab maintenance and surveillance	Clinical complete response maintained; PFS >36 months, OS >4 years	No new adverse events

**Figure 2:**
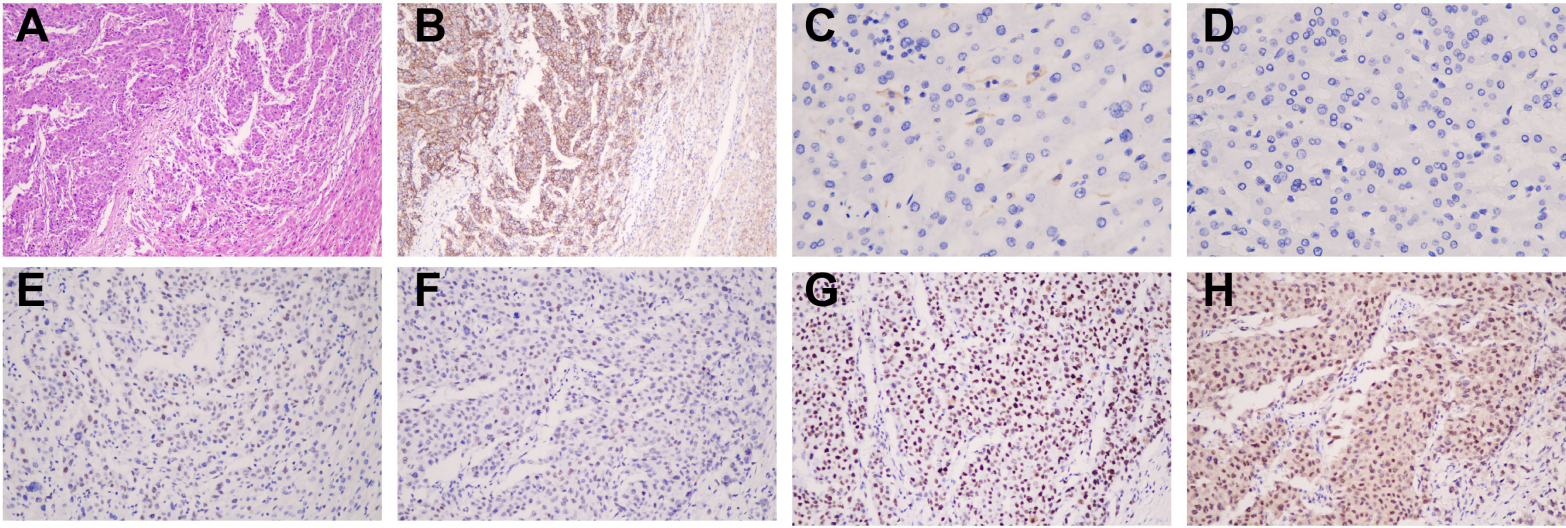
Histopathological and immunohistochemical features of the resected hepatocellular carcinoma. **(A)** Hematoxylin–eosin staining (×100) showing hepatocellular carcinoma with a macrotrabecular growth pattern, moderate differentiation, and moderate nuclear atypia. **(B)** Immunohistochemical staining for β-catenin (×100), demonstrating positive staining in the tumor cell membrane and cytoplasm. **(C, D)** Immunohistochemical staining for programmed death-ligand 1 (PD-L1) (×100), showing negative expression in the resected liver tumor specimen. **(E–H)** Immunohistochemical staining for the mismatch repair (MMR) proteins MLH1, MSH2, MSH6, and PMS2 (×200), confirming positive expression of all four proteins.

Considering the solitary 3.5-cm tumor, PVTT confined to the right portal vein branch, preserved liver function (Child-Pugh A), ECOG performance status 0, and the absence of extrahepatic metastasis, a consensus decision was made to pursue a multidisciplinary team (MDT)-guided conversion strategy aimed at curative-intent resection. Accordingly, he underwent transarterial chemoembolization (TACE) with an epirubicin (20 mg)-lipiodol emulsion on October 10, 2021, to reduce tumor burden and allow reassessment of resectability. One month later, follow-up imaging demonstrated favorable lipiodol deposition within the primary lesion, indicating a good local response. The PVTT remained confined to the right branch without progression to the main portal trunk, and no extrahepatic metastasis was detected. Based on the imaging findings and the patient’s overall clinical condition, surgical resection was recommended by the MDT. He subsequently underwent three-dimensional laparoscopic partial resection of lesions involving the right posterior and right anterior lobes, together with cholecystectomy and adhesiolysis. Pathology confirmed nodular HCC (4.2 × 4.0 × 3.8 cm, Edmondson–Steiner grade III) with a trabecular and solid pattern, extensive microvascular invasion (M2 grade), and HBV-related cirrhosis. Immunohistochemistry revealed AFP (+), ATRX (+), Glypican-3 (+), CD34 (increased vascular staining), GS (+), HBsAg (+), HBcAg (–), Hepa (–), PD-L1 combined positive score (CPS) <1, proficient mismatch repair (pMMR), and membranous β-catenin positivity (**Figure 2**). The right anterior lobe contained intravascular HCC thrombi, while the gallbladder showed chronic cholecystitis with cholesterol polyps. Surgical margins were negative. The patient was considered to be at extremely high risk of postoperative recurrence because of M2 microvascular invasion, Edmondson-Steiner grade III differentiation, cirrhosis, and intravascular tumor thrombus. Therefore, adjuvant lenvatinib (8 mg once daily) was initiated in December 2021, followed by two additional sessions of TACE on December 27, 2021, and February 28, 2022, as an individualized intensified adjuvant strategy. This decision was supported by the guideline framework and emerging evidence available at that time, as well as the patient’s favorable tolerance and early response to the initial TACE. The interval between the two TACE procedures was determined by postoperative recovery and reassessment of treatment fitness. AFP levels remained within the normal range thereafter. Post-treatment CT showed postoperative changes with residual cirrhosis, portal hypertension, and splenomegaly.

**Figure 3:**
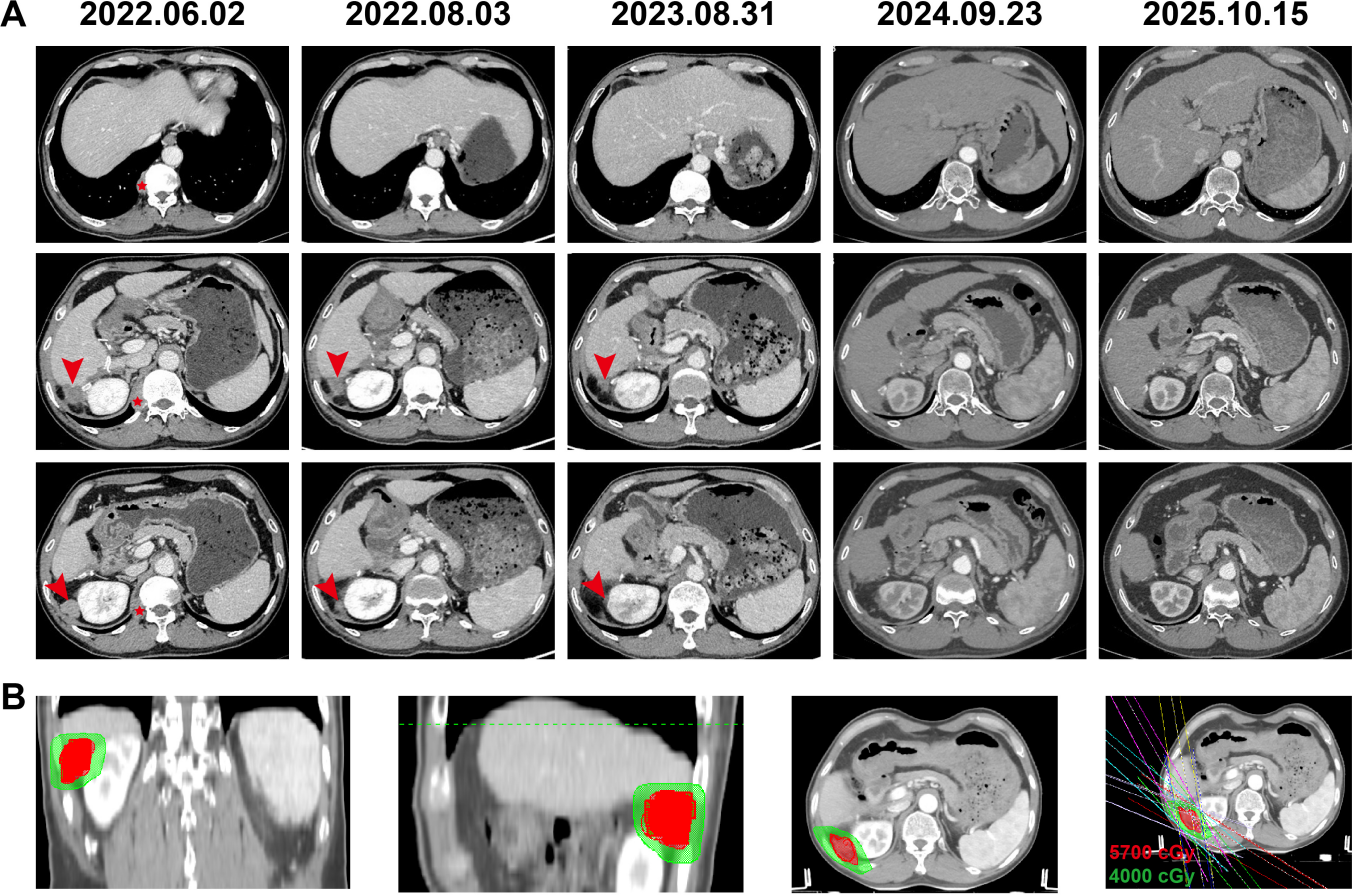
Radiologic evolution of postoperative recurrent lesions and radiotherapy planning. **(A)** Serial contrast-enhanced CT images at the time of postoperative recurrence and at subsequent follow-up time points, illustrating the radiologic response of the hepatic and extrahepatic lesions during treatment. The displayed images represent selected sections for longitudinal comparison. Arrowheads indicate the right perirenal lesion, and asterisks indicate the right subpleural metastatic lesion. **(B)** Intensity-modulated radiotherapy (IMRT) planning images in coronal, sagittal, and axial views, together with the beam arrangement and dose distribution. The gross tumor volume (GTV, outlined in red) was prescribed 5700 cGy, and the region receiving 4000 cGy is shown in green.

Six months later, the patient presented with persistent right flank pain (Numerical Rating Scale score 6). Serum AFP had increased to 1,179 ng/mL. CT identified a 2.5 × 2.2 cm enhancing nodule in the right perirenal region and a right subpleural nodule measuring 2.7 cm in its longest diameter. The subpleural nodule was considered metastatic, while the perirenal lesion was indeterminate but favored to represent metastasis, suggesting postoperative recurrent disease (**Figure 3A**). From June 9 to July 5, 2022, intensity-modulated radiotherapy (IMRT) was delivered to the perirenal lesion (57 Gy in 19 fractions) for pain control and local tumor management (**Figure 3B**). Tislelizumab (200 mg every 3 weeks) was initiated one week after radiotherapy commenced. After 15 fractions, notable improvement in pain control was observed, allowing a reduction in the oxycodone dosage. One month after radiotherapy, the patient achieved complete pain relief and was off analgesic medication, with serum AFP levels returning to normal. Follow-up contrast-enhanced CT revealed shrinkage of the perirenal lesion to 1.2 × 1.0 cm, while the non-irradiated right subpleural lesion had completely resolved. The overall response was classified as partial response (PR) by RECIST v1.1 criteria, with >70% reduction in target lesion diameters (**Figure 3A**). Two months later, repeat contrast-enhanced CT demonstrated a further decrease in the right perirenal nodule, measuring approximately 0.6 × 0.6 cm, indicating continued tumor regression (**Figure 3A**).

**Figure 4:**
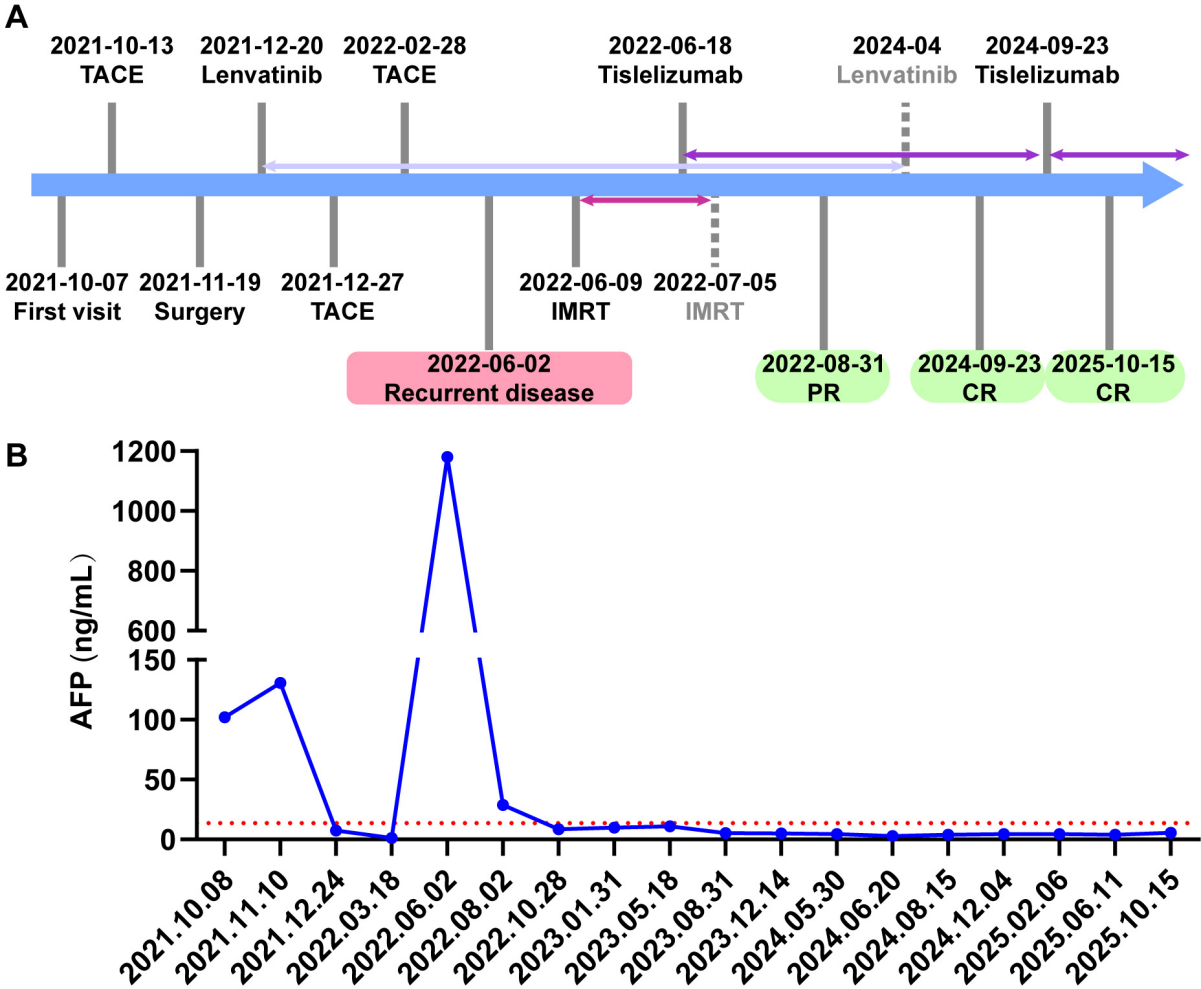
Overview of treatment course and serum alpha-fetoprotein (AFP) levels. **(A)** Treatment timeline from initial diagnosis to last follow-up. **(B)** Serum AFP levels throughout treatment. PR, partial response; CR, complete remission; TACE, transarterial chemoembolization.

Maintenance tislelizumab therapy was continued thereafter. The patient experienced no immune-related adverse events, including thyroid dysfunction, pneumonitis, myocarditis, dermatologic reactions, or hepatic toxicity. Regular follow-up imaging and AFP monitoring demonstrated sustained disease control. One year after radiotherapy, CT revealed a residual perirenal lesion of 0.6 × 0.6 cm (**Figure 3A**), which remained stable on subsequent scans. Due to stable disease and low-grade adverse events considered more consistent with lenvatinib toxicity, including grade 1 diarrhea, grade 2 hypertension, and grade 1 proteinuria (according to CTCAE v5.0), lenvatinib was discontinued in April 2024, and tislelizumab was continued as monotherapy. Two years after initiation of radiotherapy and immunotherapy, CT on September 23, 2024, showed no evidence of residual enhancing lesions in the perirenal region, consistent with clinical CR (**Figure 3A**). Cirrhosis with portal hypertension and splenomegaly remained stable. The tislelizumab dosing interval was extended to every two months. At the latest follow-up in October 2025, CT and MRI scans revealed no evidence of disease (**Figure 3A**), and AFP remained within the normal range. The patient has remained progression-free for more than 36 months, with a clinical complete response sustained for over one year since September 23, 2024, and an overall survival exceeding four years. The overall treatment timeline and corresponding changes in AFP levels are summarized in **Figure 4**.

## Discussion

This case highlights that, after disease progression following locoregional therapy, curative surgery, and TKI treatment, an immunotherapy-based multimodal approach can achieve durable and deep responses in advanced HCC. Under MDT guidance, the patient, who initially presented with BCLC stage C HCC and PVTT, underwent TACE followed by surgical resection and then received adjuvant lenvatinib together with two additional sessions of TACE as an individualized intensified anti-recurrence strategy, which normalized AFP levels. Despite this intensive multimodal approach, the patient developed a right perirenal lesion and a synchronous right subpleural metastatic lesion within six months, accompanied by a sharp rise in AFP to 1,179.7 ng/mL. Management was then shifted toward an immunotherapy-based strategy. IMRT was delivered to the perirenal lesion, and tislelizumab was initiated one week later. After completion of radiotherapy, the right perirenal lesion decreased in size, whereas the right subpleural lesion completely disappeared, resulting in a partial response. With continued tislelizumab therapy, the patient subsequently achieved a clinical complete response and has remained disease free on long-term follow-up. Since then, the patient has remained progression-free for more than 36 months, with overall survival now exceeding four years.

Treatment decision-making in HCC is increasingly complex, encompassing initial diagnosis, response evaluation, and management of progression. Although HCC with PVTT is generally classified as BCLC stage C, this group is clinically heterogeneous. According to the BCLC framework, systemic therapy should in principle be considered for patients with macrovascular invasion; however, selected patients with limited branch PVTT, preserved liver function, good performance status, and no extrahepatic metastasis may still be candidates for a more aggressive locoregional or conversion strategy (27, 28). In the present case, the patient had a solitary 3.5-cm tumor, PVTT confined to the right portal vein branch, Child-Pugh A liver function, ECOG 0, and no extrahepatic metastasis. Therefore, MDT selected upfront TACE to reduce tumor burden and reassess resectability, followed by surgery with curative intent. This strategy is consistent with the Guidelines for Diagnosis and Treatment of Hepatocellular Carcinoma with Portal Vein Tumor Thrombus in China (2021 Edition), which support resection in selected patients with type I/II PVTT, preserved liver function, and good performance status, and with prior proposals that BCLC stage C should be subclassified because patients with branch PVTT and Child-Pugh A liver function may benefit from individualized treatment allocation rather than a uniform approach.

Postoperative recurrence remains common and typically peaks within the first year after surgery (29, 30). Early recurrence is often attributed to occult micrometastases seeded by aggressive primary tumor biology (e.g., large size, multiplicity, vascular invasion, high grade, elevated AFP) and is associated with inferior long-term outcomes (31). In practice, most patients require additional therapy for recurrent or metastatic lesions, and the choice of post-recurrence strategy is as critical as initial management (29). In this patient, the recurrence risk was particularly high because of M2 microvascular invasion, Edmondson-Steiner grade III differentiation, cirrhosis, and intravascular tumor thrombus. Accordingly, in the absence of a globally established adjuvant standard at that time, postoperative lenvatinib plus additional TACE was adopted after MDT discussion as an individualized intensified strategy to reduce recurrence. This approach was supported by the 2021 Chinese PVTT guideline and by emerging retrospective evidence suggesting improved disease-free survival with adjuvant TACE plus TKI, compared with TACE alone, in high-risk HCC after hepatectomy (28, 32). The patient’s good tolerance and early response to the initial TACE further reinforced this decision. Nevertheless, although upfront locoregional therapy and surgery reduced tumor burden and removed the primary lesion, they were insufficient to eradicate micrometastatic disease, which subsequently manifested as postoperative recurrent disease with a right perirenal lesion and a synchronous right subpleural metastatic lesion.

Locoregional therapies such as TACE and hepatic arterial infusion chemotherapy (HAIC) are widely utilized in HCC and can increase resection rates when incorporated into multimodal treatment plans. TACE induces tumor hypoxia and chemotherapy-related injury that can both kill tumor cells and activate anti-tumor immunity. However, TACE-driven hypoxia may also upregulate angiogenesis and promote relapse (33). In the present case, the first post-TACE reassessment showed favorable lipiodol deposition within the primary lesion, while PVTT remained confined to the right portal vein branch without progression and no extrahepatic metastasis was detected, supporting the transition from initial locoregional treatment to surgery. TKIs (e.g., sorafenib, lenvatinib) inhibit VEGF/VEGFR-mediated pathological angiogenesis, promote vascular normalization, and can partially reverse an immunosuppressive tumor microenvironment (TME), thereby facilitating T-cell infiltration and antigen presentation. Consequently, the combination of TACE and TKI therapy may create a permissive milieu for PD-1/PD-L1 blockade to relieve immune inhibition and exert synergistic anti-tumor effects with immunotherapy (33). In the present case, the sequence of TACE, surgery, and lenvatinib before postoperative recurrent disease may have contributed to vascular normalization and antigen exposure, thereby “preconditioning” the TME and sensitizing the tumor to subsequent PD-1 inhibition.

Immunotherapy has transformed systemic therapy for advanced HCC, yet achieving a CR with PD-1 inhibitor monotherapy remains challenging. According to the American Association for the Study of Liver Diseases guidelines, ICI-based systemic therapy is recommended for patients at high risk of recurrence after curative resection or ablation (34, 35). The phase III IMbrave050 trial demonstrated that adjuvant atezolizumab plus bevacizumab significantly improved recurrence-free survival (RFS) compared with active surveillance in high-risk patients, establishing ICI-based combinations as a new standard of care (36, 37). However, not all patients have access to these regimens because of cost, availability, or comorbid conditions, underscoring the value of alternative PD-1 inhibitors such as tislelizumab, which has demonstrated durable efficacy with acceptable safety in advanced HCC. The phase II RATIONALE-208 study reported a median OS of 13.2 months and a median duration of response of 36.1 months in previously treated advanced HCC (38). The phase III RATIONALE-301 trial confirmed noninferiority to sorafenib in the first-line setting, with a median OS of 15.9 months, median PFS of 2.1 months, and an ORR of 14.3% (39). These data supported tislelizumab’s approval in China for both first- and second-line use in unresectable HCC.

Combining TKIs with ICIs appears to amplify therapeutic benefit beyond that of either agent alone. By inhibiting VEGF signaling and remodeling the abnormal vasculature, TKIs such as lenvatinib can alleviate hypoxia, promote effector T-cell infiltration, and shift the TME toward a more inflamed phenotype (21, 40). In the CARES-310 trial, camrelizumab plus apatinib achieved a median OS of 22.1 months compared with 15.2 months for sorafenib, particularly benefiting patients with HBV-related HCC (20). A large real-world study from China reported that lenvatinib combined with PD-1 inhibitors, including tislelizumab, achieved favorable outcomes, with a median overall survival of 17.8 months, progression-free survival of 6.9 months, an objective response rate of 19.6%, and a disease control rate of 73.5% (23). Furthermore, a retrospective study in Chinese BCLC-C HCC patients progressing after lenvatinib showed that adding tislelizumab prolonged median PFS (6.8 vs. 4.5 months) and OS (14.0 vs. 10.4 months) compared with tislelizumab monotherapy, especially in Child-Pugh A patients (41). Consistent with these findings, our patient demonstrated a remarkable clinical response to tislelizumab after receiving TACE, surgical resection, and lenvatinib therapy. The preceding locoregional and TKI treatments may have normalized tumor vasculature, enhanced antigen presentation, and promoted immune cell infiltration, thereby sensitizing the tumor to immunotherapy. This case further supports the clinical potential of tislelizumab-based multimodal therapy to achieve durable remission and prolonged survival in previously treated HCC.

Radiotherapy and immunotherapy have emerged as complementary modalities with potential synergistic anti-tumor effects (24). Beyond direct cytotoxicity, radiotherapy induces immunogenic cell death and enhances neoantigen presentation, promoting T-cell infiltration and activation. These radiation-induced immune responses can extend beyond the irradiated field to induce abscopal tumor regression, an effect that may be further strengthened by PD-1/PD-L1 blockade (42). Preclinical and clinical evidence suggest that the timing of combination therapy is critical. Evidence suggests initiating immune checkpoint blockade during or shortly after radiotherapy (less than 7 days) maximizes therapeutic synergy, whereas delayed administration may lead to T-cell exhaustion (43–45). In this case, tislelizumab initiated one week after IMRT was associated with regression of both irradiated and non-irradiated lesions, suggesting a synergistic systemic immune effect. During more than three years of follow-up, the patient remained free of recurrence. This outcome suggests that IMRT combined with PD-1 blockade, following prior TKI-mediated vascular normalization, can lead to durable systemic disease control.

While immunotherapy-based combinations have significantly advanced HCC management, their clinical benefit remains confined to a subset of patients. The heterogeneity of tumor biology, etiologic diversity, and the liver’s unique immune milieu hinder accurate identification of responders, underscoring the need for reliable predictive biomarkers (46). PD-L1 expression assessed by immunohistochemistry has shown variable associations with response to PD-1 blockade across cancers (47). In KEYNOTE-224 trial, higher PD-L1 CPS correlated with pembrolizumab response in some HCC patients progressing after sorafenib (48). However, PD-L1 expression in HCC is highly heterogeneous and inconsistently predictive (49, 50). Tumor mutational burden (TMB) is generally low in HCC and likewise has limited prognostic or predictive utility (51). In our case, despite a PD-L1 CPS < 1% indicating a “cold” immune phenotype, the patient achieved a durable and robust response to tislelizumab-based multimodal therapy. This finding suggests that PD-L1 expression alone cannot reliably predict ICI responsiveness in HCC and highlights the urgent need to identify additional biomarkers to guide immunotherapy selection.

The optimal duration and scheduling of maintenance immunotherapy in HCC remain undefined, particularly for patients who achieve CR. In this case, lenvatinib was discontinued after two years, whereas tislelizumab monotherapy has been continued for three years, during which the patient has remained free of disease. Limited data suggest that continuing immunotherapy for at least six months after achieving CR may reduce recurrence risk, but robust prospective evidence is lacking (52). Establishing consensus on the optimal timing and duration of immunotherapy in HCC is urgently needed. In the future, emerging techniques such as circulating tumor DNA (ctDNA) and circulating tumor cell (CTC) monitoring may potentially help guide decisions on discontinuing immunotherapy and support individualized follow-up strategies. Further studies are also warranted to define the most appropriate stage for introducing immunotherapy and to optimize its integration within multimodal treatment frameworks for HCC. For those with disease progression or limited response to first-line antiangiogenic or immunotherapy-based regimens, exploring effective second-line or alternative treatment approaches will be essential to improve long-term outcomes. After detailed discussion with the multidisciplinary team, the patient remained adherent to the planned multimodal therapy and follow-up schedule. He was encouraged by the sustained disease control achieved during treatment and expressed satisfaction with the overall outcome.

In summary, this case demonstrates that an immunotherapy-centered multimodal strategy can achieve clinical complete remission and long-term survival in a patient with BCLC stage C hepatocellular carcinoma who developed extrahepatic metastases after surgery and lenvatinib treatment. The sustained CR and extended PFS/OS observed here highlight the potential of tislelizumab-based combination therapy to extend therapeutic benefit to patients with complex, previously treated HCC and underscores the importance of integrating systemic immunotherapy into individualized, multidisciplinary management.

## Data Availability

The original contributions presented in the study are included in the article/supplementary material. Further inquiries can be directed to the corresponding authors.
